# Clinical genome sequencing and population preferences for information about ‘incidental’ findings—From medically actionable genes (MAGs) to patient actionable genes (PAGs)

**DOI:** 10.1371/journal.pone.0179935

**Published:** 2017-07-03

**Authors:** Thomas Ploug, Søren Holm

**Affiliations:** 1 Aalborg University Copenhagen, Centre for Applied Ethics and Philosophy of Science, Department of Communication, Kbh. SV, Denmark; 2 University of Manchester, Centre for Social Ethics and Policy, School of Law, Manchester, United Kingdom; 3 Center for Medical Ethics, Faculty of Medicine, University of Oslo, Oslo, Norway; 4 Centre for Applied Ethics, Aalborg University, Aalborg, Denmark; Ohio State University Wexner Medical Center, UNITED STATES

## Abstract

Whole genome or exome sequencing is increasingly used in the clinical contexts, and ‘incidental’ findings are generated. There is need for an adequate policy for the reporting of these findings to individuals. Such a policy has been suggested by the American College of Medical Genetics and Genomics (ACMG). We argue that ACMG’s policy is overly paternalistic, and that an adequate policy must take into account population preferences. We conducted a choice based conjoint survey of population preferences for reporting in a representative sample of the Danish population. In a 12 task survey respondents were asked about their preference for reporting in relation to three scenarios with seven different attributes. Of 1200 respondents 66.4% participated. We show that population preferences for reporting differs from ACMG’s recommendations, and suggest a new policy based on both medically and patient actionable genes.

## Introduction

Whole genome or exome sequencing (WGS/WES) will be increasingly applied in everyday clinical practice for at least three reasons: 1) it is necessary for precise diagnostics, prognostics and personalised medicine (e.g.pharmacogenomics), 2) it is becoming as cheap and fast as targeted genetic analysis, and 3) there is significant utility in storing and potentially reusing the genetic sequence. However, the increased use of genome sequencing is generating large amounts of incidental findings of pathogenic variants. As has been widely recognised there is need for a policy that adequately deals with the reporting of these findings to patients.

In 2013 the American College of Medical Genetics and Genomics (ACMG) suggested a policy for the reporting of incidental findings consisting of a minimum list of findings to be actively sought and reported to an individual. The criteria for entering the list included ‘in most cases’ 1) that the variant was a recognised or expected cause of disorder, and 2) that ‘confirmatory approaches for medical diagnosis would be available’. Priority was given to pathogenic variants associated with disorders 3) ‘for which preventive measures and/or treatments were available’, and 4) disorders in which individuals may ‘be asymptomatic for long periods of time’. Finally, ACMG recommended 5) ‘that only variants with a higher likelihood of causing disease’ should be reported. [1,2] The ACMG approach raises two problems: 1) It ignores the possibility that patients may have an interest in not knowing about genetic risk factors or only know of them selectively. 2) It ignores the possibility that patients’ interests in information about genetic risk factors may not be limited to what ACMG calls ‘medically actionable genes (MAGs)’. [3] The right not to know has been widely discussed, [3–11] and the ACMG has in a recent update of their recommendations included the option of opting out of genetic analysis entirely. [12]

We believe that a policy for reporting of incidental findings solely taking into account professional standards is overly paternalistic, and that an adequate policy should take into account individuals’ interests in the form of population preferences. The original ACMG policy was developed in relation to the very early phase of the introduction of genome sequening into clinical practice and this may have influenced the policy development by creating a focus on what was seen as the major problem cases. The available data about patient and population preferences would also have been limited. This, together with the focus on the professional point of view probably explains the restrictive nature of the policy, but it may now be time to move beyond it.

There is, however still limited evidence on population preferences for reporting of incidental findings (we review and discuss the existing literature in more detail in the Discussion, below). And, more importantly, we have found very little systematic evidence on how a population weigh factors relevant for reporting of incidental findings relative to each other. We have found one conjoint analysis study of preferences for the return of research results in the research context [13], and one study developing a discrete choice experiment (DCE) instrument to measure preferences for return of information from clinical sequencing. [14]

We have therefore studied the relative importance of different aspects of genetic findings influencing preferences for information about incidental findings in the Danish population, and on this basis we suggest a new policy for reporting based on a combination of medically and patient actionable genes (MAGs and PAGs).

## Materials and methods

We designed and performed a choice based conjoint (CBC) survey in the general Danish population. Attributes and levels for the CBC survey are based on prior exploratory focus groups.

### Focus groups

Three focus groups were held in different parts of Denmark each with 7–8 participants drawn from TNS Gallup’s Danish consumer panel. A short introduction to relevant facts about genetics was presented and the group then asked to sequentially discuss three different scenarios in which ‘incidental findings’ can occur: 1) Pharmacogenomic analysis to decide choice of therapy, 2) genetic analysis in relation to possible familial cancer, 3) research on tissue donated for research. In relation to each scenario the groups was asked to identify factors concerning an ‘incidental finding’ that would make them want to have information about the finding, and when all factors had been identified to rank these in order of importance. Following the discussion of the three scenarios the group was then presented with a list of factors that have been mentioned in the literature as important for feedback decisions [15–23], and the group was asked to place the factors identified in the group and in the literature into three categories of importance (very important, important, not important). Focus groups were audio-recorded, but not transcribed.

The analysis of the focus group data showed that seven factors were consistently mentioned as important, and these were chosen as the attributes for the CBC survey:

1. Scientific certainty of relation between genetic finding and disease
2. Risk of developing disease given genetic finding
3. Severity of disease
4. Possibility of treatment
5. Possibility of prevention
6. Time between finding and first clinical manifestation of disease
7. Reproductive relevance

For each attribute a number of levels were developed based on the focus group discussions. The final level descriptions were developed in collaboration with the survey quality control unit of TNS Gallup.

### Design of CBC survey

The CBC survey was designed, run and analysed using the Sawtooth suite of programs. [24]

**Fig 1:**
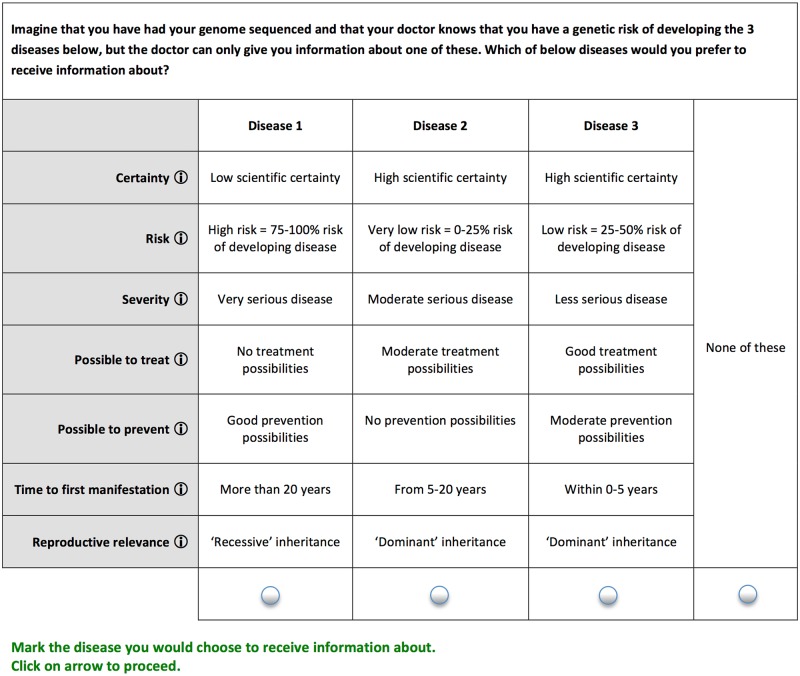
Choice-task. An example of a choice-task with 3 concepts.

For each of the seven identified attributes a number of levels were described (see Table 1). A ‘concept’ is a combination of a level for each of the seven attributes and respondents were presented with 12 choice tasks between three concepts and a ‘none of these’ option. An example is shown in Fig 1 below.

**Table 1:** Importance of attributes and parth-worth-utilities of levels.

Attribute	Importance*	Levels and part-worth utilities**	
**Scientific reliability**	12.8%	High scientific reliability	36.8
		Low scientific reliability	-36.8
**Risk of developing disease**	19.1%	Very small risk 0–25%	-58.8
		Low risk 25–50%	-16.8
		Moderate risk 50–75%	28.5
		High risk 75–100%	49.2
**Severity of disease**	21.9%	Very serious disease	65.4
		Moderately serious disease	0.8
		Less serious disease	-66.3
**Possibility of treatment**	10.8%	Good treatment possibilities	16.8
		Moderate treatment possibilities	-3.9
		No treatment possibilities	-12.9
**Possibility of prevention**	15.2%	Good prevention possibilities	38.2
		Moderate prevention possibilities	6.6
		No prevention possibilities	-44.7
**Time to first manifestation**	13.5%	0–5 years	26.8
		5–20 years	5.7
		>20 years	-31.5
**Reproductive relevance**	6.7%	‘Dominant’ inheritance	15.7
		‘Recessive’ inheritance	-15.7

* The importance of an attribute is the overall weight that respondents on average place on that attribute in their decisions taken as a percentage of the complete weight of all attributes.

** A positive part-worth utility indicate that a level of an attribute makes it more likely that a respondent would wish to have information about a finding, and the conversely for a negative part-worth utility. For any given attribute the part-worth utilities sum to zero.

The choice sets were generated using the complete enumeration method in the Sawtooth SSI Web (version 7.0.30) module. [25] The complete enumeration method generates CBC designs conforming to the principles of 1) Minimal Overlap of attribute levels within a single choice task, 2) Level Balance across the set of choice task presented to each respondent, and 3) Orthogonality, i.e. the levels of different attributes are chosen independently. 100 unique sets of CBC questionnaires were generated each of which was presented to an approximately equal number of respondents.

The choice to be made was described as: “Imagine that your genes have been mapped and that the doctor knows that you have a genetic risk for the 3 diseases below, but the doctor is only allowed to give you information about one of the diseases. Which of the diseases below would it be most important for you to get information about?” For each of the attributes a ‘pop up’ provided a definition of the attribute and a description of the levels.

### Demographic data

The questionnaire included questions about a range of demographic data, including questions about knowledge about genetics, and hereditary or chronic disease in the family.

### Sample

A stratified sample of 1200 potential participants was drawn from TNS Gallup’s Danish consumer panel of 53,000 active members. The size of the sample was not based on a power calculation, since there are no prior data from which we could estimate the likely variation in answers. The sample size is therefore based on the marketing industry rule of thumb that the number of respondents should be approximately 100 x the number of attributes [24]. The TNS Gallup panel is recruited to be representative of the Danish adult population. E-mails were sent to the potential participants inviting them to participate in the study. After 3, 11 and 29 days non-responders, i.e. those who had not completed the questionnaire or who had not visited the web-site hosting the questionnaire were reminded by e-mail. After 6, 22 and 31 days non-responders were contacted by phone.

### Statistical analysis

Part-worth utilities were estimated using Sawtooth CBC/HB (version 5.5.3) to perform a hierarchical Bayes method estimation, running 190,000 ‘burn in’ iterations and 10,000 draws per respondent. A detailed description of the hierarchical Bayes method and its implementation can be found at [26]. The manual is freely available online.

Latent class analysis was performed using the LC module in the Sawtooth suite of progams. We ran analysis for solutions with 2–6 segments, with 5 replications, and a maximum of 100 iterations. [27] All solutions converged within 100 iterations. The best solution was the five segment solution reported in the results.

Demographic data, part-worth utilities and answers to non-CBC questions were imported to IBM SPSS Statistics (version 20). Demographic data were tabulated, and univariate relationships between respondent characteristics and latent class membership were analysed using the Goodman and Kruskal tau statistic in the crosstabs function. Univariate relationships between respondent characteristics and attribute importance were analysed using ANOVA.

The dataset is available in Excel format in S1 File.

### Ethics

Focus groups and anonymous questionnaires with participants from the general public do not require approval from a Research Ethics Committee or similar body in Denmark.

## Results

Of the 1200 potential respondents contacted, 1048 opened the link to the questionnaire, 797 completed it fully and 168 completed it partially. The analysis is based on the 797 complete answers giving a response rate of 66.4%. A sample efficiency analysis was performed comparing respondents to the desired sample characteristics in relation to gender, age, geographical region, and level of education (SoS 33.78, df 785, Efficiency 98.45%).

387 (48.6%) respondents were men and 410 (51.4%) were women. The median age was 47.8 years with a quartile range of 33.5–63.1 years. The highest educational attainment was School or High School 244 (30.6%), Further education 252 (31.6%), and University 252 (31.6%). 35 (4.4%) respondents stated that their knowledge about genetics was ‘high’, 235 (29.5%) that they knew ‘some’, 370 (46.4%) that they knew ‘little’, 139 (17.4%) that they knew ‘nothing’, and 19 (2.4%) answered ‘don’t know’. 301 (37.8%) stated that they had a hereditary condition in the family and 265 (33.2%) that they had a chronic disease in the family.

The results of the conjoint analysis are presented in Table 1.

The conjoint analysis shows that the importance ranking of the attributes influencing the preference for feedback is Severity of disease, Risk of developing disease, Possibility of prevention, Time to first manifestation, Scientific reliability, Possibility of treatment, Reproductive relevance.

The results of the latent class solution are found in Table 2.

**Table 2:** Latent class solution.

Latent class		1	2	3	4	5
**Total N = 797**	**n**	399	189	80	43	85
**Attribute**	**%**	50.10%	23.70%	10.00%	5.40%	10.70%
**Scientific reliability**	**High scientific reliability**	28.16	46.86	45.57	19.10	147.25
	**Low scientific reliability**	-28.16	-46.86	-45.57	-19.10	-147.25
**Risk of developing disease**	**Very small risk 0–25%**	-88.72	-20.35	-64.13	-12.00	-78.40
	**Low risk 25–50%**	-34.50	-5.39	-24.98	28.84	-13.99
	**Moderate risk 50–75%**	41.05	23.26	32.81	-23.42	31.26
	**High risk 75–100%**	82.17	2.47	56.29	6.57	61.13
**Severity of disease**	**Very serious disease**	120.02	6.95	78.30	-172.86	41.96
	**Moderately serious disease**	3.34	-6.44	-8.07	107.06	12.28
	**Less serious disease**	-123.36	-0.50	-70.23	65.79	-54.25
**Possibility of treatment**	**Good treatment possibilities**	1.92	108.03	18.61	28.30	29.50
	**Moderate treatment possibilities**	-3.56	-33.36	-5.36	5.53	-5.36
	**No treatment possibilities**	1.63	-74.66	-13.25	-33.84	-24.13
**Possibility of prevention**	**Good prevention possibilities**	38.99	137.46	69.36	45.74	33.97
	**Moderate prevention possibilities**	7.59	-27.74	5.87	8.35	9.39
	**No prevention possibilities**	-46.59	-109.72	-75.24	-54.10	-43.37
**Time to first manifestation**	**0–5 years**	45.50	-32.57	55.52	72.80	11.85
	**5–20 years**	8.65	8.20	5.37	-21.38	-2.80
	**>20 years**	-54.16	24.36	-60.89	-51.42	-9.05
**Reproductive relevance**	**‘Dominant’ inheritance**	19.30	31.21	23.49	-21.67	8.91
	**‘Recessive’ inheritance**	-19.30	-31.21	-23.49	21.67	-8.91
	NONE	-496.74	-994.26	88.81	525.14	-138.24

The latent class analysis identifies 5 groups of respondents with distinct patterns of attribute weightings. The largest group (50.1%) has the same pattern of weightings as the overall conjoint analysis, the second largest group (23.7%) sees prevention and treatment possibilities as very important, a third group (10.7%) weighs all attributes as important but does not attach much weight to any of them, the fourth group (10.0%) sees severity of disease as a factor counting against providing feedback, and the fifth and smallest group (5.4%) puts great emphasis on scientific reliability.

The analysis of the univariate relationships between respondent characteristics and latent class membership shows statistically significant, but weak relationships between latent class and age, stated level of knowledge of genetics, and the presence of hereditary disease in the family (dns). We also performed the analysis for gender, level of education and chronic disease in the family, but found no statistically significant relations.

The analysis of the univariate relationship between respondent characteristics and the importance given to individual attributes is shown in Table 3.

**Table 3:** Respondent characteristics and the importance of attributes.

Attribute	Gender	Age	Level of education	Self-rated knowledge of genetics
**Scientific reliability**	* More important for males		* More important for groups with higher level of education	
**Risk of developing disease**				* More important for groups with low self-rated knowledge
**Severity of disease**		*** More important for younger groups		*** More important for groups with high self-rated knowledge
**Possibility of treatment**		*** More important for older groups	* More important for groups with lower level of education	
**Possibility of prevention**			** More important for groups with higher level of education	
**Time to first manifestation**				
**Reproductive relevance**				

* p<0.05

** p<0.01

*** p<0.001

We also performed the analysis for hereditary disease and chronic disease in the family but found no significant relationships. It is interesting to note that there are no statistically significant relations between gender or age and the importance given to the attribute of ‘Reproductive relevance’.

## Discussion

### Limitations of current study

The response rate of 66.4% is good for a population survey, and the sample efficiency analysis shows that the respondents are very similar to the complete sample in relation to the stratification variables. There is thus some reason to think that the findings reflect the views in the general Danish population.

The choice situation is hypothetical but is in many ways similar to the situation that patients would be in when contemplating WGS/WES for pharmacogenomics. The doctor suggesting sequencing would usually not be a specialist in genetics and would not be able to provide much specific advice on what incidental findings could occur.

The question of whether patients should be able to opt-out of being informed about MAGs is controversial, but it is outside the scope of the present study. The latent class analysis does, however indicate that there may be a small sub-section of the population who has a strong preference for not being informed about some genetic findings. For most respondents the fact that a disease is severe is a reason to want feedback, but for some it is a very strong reason to not want feedback, and the weightings indicate that it is such a strong reason that it outweighs considerations of treatability and/or preventability for this sub-group (latent class 4).

This study has been conducted in the Danish population and we use the results to problematize what on the face of it is a US only policy, i.e. the MAG based policy of the ACMG. We think this is warranted for two reasons 1) ACMG policies are discussed and influence policy making around the world, and 2) even though the exact weighting of the attributes of genetic findings is likely to differ between countries the present study shows that they may diverge significantly from professional opinion, and this is also likely to be the case in the USA.

### Previous empirical research on preferences for reporting of incidental findings

Previous empirical research on criteria for reporting of incidental findings in research or clinical genetics settings have mainly studied the views of health care professionals including in particular health care professionals directly involved in genetics, and the views of IRB chairs. [28–45] A commonly shared view among the professionals is that actionability in terms of treatability and preventability is a key criterion for reporting. [28,30,31,33,34,36,40,42–44] A number of studies have examined the general public’s view of criteria for reporting incidental findings. [13,30,34,44,46–51] These studies commonly find that the actionability and the seriousness of the diseases associated with incidental findings are of importance to the public. A few studies indicate that both among health professionals and the public there is a wish for individualised reporting of incidental findings. [42,45,49,50]

Methodologically most studies either apply a qualitative research design based on various forms of individual or focus group interviews [28–30,32,34,35,38–40,45,47,49,50] or a quantitative research design based on surveys that directly ask respondents to state their preferences for reporting. [31,33,36,37,42–44,46,48,51]. We have identified a single study applying conjoint analysis [13]. The current study differs from this study with respect to both the hypothetical context of reporting and the attributes and levels of the survey. The prior study investigates preferences for policies for incidental finding reporting in a research context and the choice presented is which of two research biobanks, with different policies a respondent would choose to participate in. Because of the focus on policy, the levels of those attributes where there is overlap with our study (results returned for serious diseases and results returned for treatable diseases) are stated dichotomously as either feedback or no feedback, whereas we differentiate more in relation to severity of disease and in relation to the degree of treatability.

There is one DCE study of preferences for the return of incidental findings from clinical genome sequencing in the Canadian general population and which is directly comparable to our study. [14] The study uses 5 attributes, Disease risk, Disease treatability, Disease severity, Carrier status, and Cost to you. The first 4 of these attributes are similar to our attributes of Risk, Possibility of treatment, Severity of disease, and Reproductive relevance. The last attribute, Cost to you would be irrelevant in the Danish setting. The results of this study are in many ways similar to our results. Participants expressed strong positive preferences for feedback of findings relating to high risk disorders, irrespective of possibility of treatment. There was also, as in our study significant heterogeneity of preferences, for instance in relation to the feedback of carrier status.

Our results are consistent with the previous findings in the literature, but they add significantly to knowledge by quantifying the strength of population preferences. We also show through the latent class analysis that there is considerable heterogeneity in preferences, but that this heterogeneity is not easily explainable by demographic characteristics.

### Implications for feedback policy

The population preferences for reporting of ‘incidental’ findings clearly differ from ACMG’s criteria for reporting. The ACMG criteria give priority to pathogenic variants for which preventive measures and/or treatments are available. The population prefers reporting based on the severity and likelihood of the resulting disease, and gives less weight to the existence of preventive measures and treatment.

We believe that the population’s preferences for reporting provide a reason to move beyond ACMGs recommendations. Our results show that individuals have a strong interest in gaining information about genetic factors influencing their future health in some situations where the genetic findings do not count as MAGs. Patients’ interests simply are not limited to MAGs. And, respecting people and protecting their autonomy is in part a matter of protecting their interests as defined by themselves. Our findings are not surprising since information about genes that are highly likely to result in severe disease may—medically actionable or not—be of significant relevance for an individual’s future life plans. Protecting autonomy is also a matter of protecting peoples’ ability to form, plan and pursue their own goals.

This may seem to suggest a feedback policy based on individual preferences. Every individual should get exactly the feedback he or she wants. Completely individualised reporting could be based on an individual choosing from a complete list of pathogenetic variants associated with disease. With limited knowledge about pathogenic variants, such a choice would have to be preceded by extensive counselling in order for individuals to be able to make an informed choice about their interest in the information generated through WGS/WES—not least considering that individuals could potentially decline information about medically actionable genes. Without restrictions on the number of pathogenic variants picked out for reporting, such a policy would also require extensive reporting and counselling after the WGS/WES in order for individuals to understand and be able to act on their particular genetic make-up. In a future clinical context in which genome sequencing has become part of routine diagnostic procedures, 1) it will be practically impossible to provide such extensive counselling and reporting before and after WGS/WES, 2) it would introduce what may be argued to be unjustifiable costs, and 3) it is likely to lead to ‘information overload’ undermining an individual’s understanding and ability to act on the information both in choosing the pathogenic variants for reporting and in the attempt to understand and act on the actual reporting following WGS/WES. Completely individualised reporting will therefore not be practically possible, and it could also undermine the very autonomy that it is supposed to protect.

A policy for reporting of incidental findings must take individual preferences into account without being purely individualistic, but what role is there to play in such a policy for the professional standards as captured in the ACMG policy? Professional standards for reporting cannot be ignored for two reasons: 1) Withholding information about medical actionable genes puts people at risk and may reasonably be considered to be a violation of the principle of non-maleficence and of professional obligations, and 2) by reporting incidental findings on medically actionable genes the professionals secure a transparency in the relationship to the patient that is protective of both patient and professional.

For these reasons we suggest a general reporting policy based on a combination of population preferences and professional standards. More specifically, based on our results we suggest that the list of pathogenic variants for which reporting should be offered should include both MAGs and PAGs and that a PAG would be any variant with the following characteristics:

1. The variant is associated with a moderate or high risk (>50%) of causing severe disease,
2. There is a high level of scientific evidence for the association, and
3. The manifestation of the disease is in the near to medium future.

The third criterion may be considered counterintuitive since it entails that the finding of a variant associated with a high risk of causing severe disease at the age of 40 should not be reported to a person whose genome is sequenced at the age of 20. It therefore warrants further investigation of population preferences in relation to the disclosure of significant risks in the distant future, and the criterion may have to be modified.

A policy based on these criteria will not be set in stone. It may have to be modified in the future for pragmatic or ethical reasons. Given that the number of reportable variants will increase as the scientific evidence increases it may become pragmatically impossible, within the resource constraints of a particular health care system to feed back information to an increasing number of patients. Ethical issues may also arise if individual patients have to be informed about several genetic variants, since it may be difficult for them to process the information. We believe, however, that in the current circumstances a list of pathogenic variants based on MAGs and PAGs would be actionable by the patient and at the same time manageable in the clinical context.

### Deciding policy on the basis of population preferences

Deciding a policy for reporting of incidental findings on the basis of population preferences is open to several objections: 1) It is unreasonable because it develops a policy for reporting standards on the basis of individuals’ speculation about their preferences in a hypothetical situation, 2) the policy does not take into account that within a given population individual preferences for reporting may differ over the course of time, different sub-groups in the population may have different preferences, and different populations may differ in their preferences both concerning the factors they take to be relevant for reporting and the relative weighting of these.

In the discussion of the concept of Quality Adjusted Life Years (QALY) it has been argued that the attempt to determine a score for the quality of life of different states of health based on the population’s preferences for these states is flawed because it requires for the healthy part of the population to state a preference for a state that they are not in and do not know, and for which they may have radically different preferences, if they were in it and knew it. [52] The policy suggested here does not fall prey to this objection because it is not based on individuals’ speculation about a health-state or situation that they are not in. On the contrary, we ask people to state their preferences in relation to a state that they are actually in. All people have genetic variants that they are currently unaware of.

As for the second objection, we completely agree with the observations and have also ourselves shown that sub-group differences exist. Our response is that a policy based on population preferences requires a delicate balancing between sensitivity to individual preferences and practicability, and it will not only have to be fine-tuned over the course of time but also adjusted to the specific cultural setting in which it is to be implemented. This could and should involve extensive research into the preferences of the relevant population.

## Supporting information

S1 File S1 Genomic DCE.xls. Dataset in Excel format.
(XLS)
